# Influence of gestational diabetes mellitus on the cardiovascular system and its underlying mechanisms

**DOI:** 10.3389/fendo.2025.1474643

**Published:** 2025-05-16

**Authors:** Ze Zhang, Yumeng Zhang, Shuai Huang, Min Li, Lingjun Li, Linglu Qi, Yun He, Zhice Xu, Jiaqi Tang

**Affiliations:** ^1^ Institute for Fetology, The First Affiliated Hospital of Soochow University, Suzhou, China; ^2^ Ultrasound Department, The Fourth Affiliated Hospital of Soochow University, Suzhou, China; ^3^ Department of Gynecology and Obstetrics, The First Affiliated Hospital of Soochow University, Suzhou, China; ^4^ Obstetrical Department, Women’s Hospital School of Medicine Zhejiang University, Hangzhou, China; ^5^ Department of Gynecology and Obstetrics, Taixing People’s Hospital, Taixing, China; ^6^ Research Institute for Reproductive Health and Genetic Diseases, Maternity and Child Health Care Hospital of Wuxi, Wuxi, China

**Keywords:** cardiovascular system, gestational diabetes mellitus, progeny, umbilical-placental circulation, mother

## Abstract

Gestational diabetes mellitus (GDM) is one of the most common endocrine-related complications during pregnancy, and its prevalence has increased over the past three decades. GDM adversely affects the maternal cardiovascular system, umbilical–placental blood perfusion, and fetal blood flow. We conducted a comprehensive literature search and systematically evaluated and synthesized cardiovascular changes in the mothers, umbilical–placental circulation, and the progeny following exposure to GDM. Multiple pathophysiological mechanisms underlying cardiovascular alteration were investigated, including endothelial dysfunction, insulin resistance, oxidative stress, ion channel abnormalities, inflammation, angiogenic imbalance, and epigenetic modifications. These findings provide valuable insights for developing early intervention strategies and therapeutic approaches to mitigating cardiovascular risks in both mothers and offspring following GDM exposure.

## 1 Introduction

Gestational diabetes mellitus (GDM) represents the most prevalent metabolic and endocrine disease during pregnancy, affecting approximately 20% of pregnant women in Southeast Asia (1). GDM significantly contributes to increased perinatal morbidity and elevates the risks of adverse outcomes for both mothers and their offspring. The developmental origins of cardiovascular diseases have gained increasing recognition, with numerous studies demonstrating GDM-associated cardiovascular alterations in the mothers and offspring (2, 3). The impacts of GDM on the cardiovascular system and its potential underlying mechanisms have been extensively investigated through both clinical studies and experimental research using animal models. The umbilical–placental circulation, which serves as a crucial link between mother and fetus under GDM conditions, has emerged as a focal point in contemporary research. With the rising global prevalence of GDM, there is a corresponding increase in the risks of GDM-associated cardiovascular complications in both mothers and offspring (4). It is of significant scientific and clinical importance to synthesize existing studies on cardiovascular changes and their underlying mechanisms in the mothers and offspring exposed to GDM, which would enhance our understanding of GDM-induced cardiovascular pathophysiology and potentially identify novel approaches for early prevention and treatments of these disorders. Drawing upon an extensive body of literature, this review firstly exhibited the structural and functional alterations in the cardiovascular system among the mothers, offspring, and umbilical–placental circulation following GDM exposure.

## 2 Changes in the cardiovascular system after exposure to GDM

### 2.1 Cardiovascular changes in the mothers

Comprehensive analyses have demonstrated a significant positive association between GDM and cardiovascular diseases (CVDs) (2, 5). Pregnant women with GDM have a higher risk of developing pregnancy-induced hypertension or preeclampsia than those without GDM (6). Accumulating evidence indicates that women with a history of GDM have an elevated risk of developing cardiovascular complications, including coronary artery disease, atherosclerosis, myocardial infarction, ischemic stroke, peripheral artery disease, and heart failure later in life (7–9). These associations will be reviewed in the following sections.

#### 2.1.1 Heart

Women with current or previous GDM have been demonstrated to exhibit subclinical cardiac dysfunction. Clinical studies have revealed that women with current or previous GDM have significant impairments in systolic function and diastolic function of the left ventricle, characterized by decreased global longitudinal strain (GLS, whether endocardial GLS or epicardial GLS) and an increased mitral valve E/E′ ratio (10–12). Cardiac output, ejection fraction, ventricular mass, heart rate, and stroke volume remained unchanged in women with GDM during the second and third trimesters of pregnancy (11, 13, 14). However, in women with a history of GDM, cardiac output and stroke volume were lower, while ejection fraction was higher than that in the control group (12, 15). There were reduced GLS, myocardial deformation, end diastolic/systolic volume, and pulmonary acceleration time in the right ventricle of women with GDM (13, 16), demonstrating the impact of GDM on cardiovascular function. The majority of existing studies have consistently demonstrated that volume, area, contraction function, and ejection fraction of the left atrium were not significantly changed in women with GDM throughout pregnancy (10, 11). Only a few studies have reported either unchanged or decreased left atrial reservoir and conduit strain in women with GDM (17). GDM pregnancies have shown a deterioration of the entire process of ventricular depolarization and repolarization, including increased QT dispersion and a shortened QRS complex (18). Women with a history of GDM demonstrated significantly reduced coronary flow velocity reserve values compared to healthy controls (12). In general, the observed cardiac changes in women with current or previous GDM did not meet established diagnostic criteria for clinical cardiac dysfunction in adults, which were classified as subclinical cardiac abnormalities.

#### 2.1.2 Blood pressure

Women with current or previous GDM are more likely to develop hypertension. Numerous studies have demonstrated that women with current or previous GDM exhibited elevated systolic blood pressure and mean arterial pressure (10, 13, 15). However, this finding remains controversial, as some studies have reported no significant differences in blood pressure during pregnancy and postpartum (11, 19, 20). Compared to the control group, peripheral vascular resistance in women with current or previous GDM remained increased and unchanged (10, 14). These discrepancies might be attributed to variations in study population characteristics, such as gestational weeks (GWs) and sample sizes. Asma et al. reported that among 6,841 pregnancies, the 105 cases who developed GDM had significantly higher systolic blood pressure after adjustment for maternal characteristics during GW 11–13, which might serve as a potential predictor for GDM diagnosis (21).

#### 2.1.3 Uterine artery

The uterine artery plays a crucial role in supplying blood flow to the developing fetus throughout gestation. The pulsatility index has shown inconsistent patterns when comparing GDM with normal pregnancies, including a decrease, an increase, and no changes (19, 22, 23). The resistance index was elevated in GDM pregnancies and leptin-mutation-developed GDM mice (24, 25). Compared to the control group, the ratio of peak systolic velocity to end-systolic blood flow velocity and blood flow were remarkably higher in GDM pregnancies during GW 24–31 (24), while both peak systolic velocity and diastolic velocity were significantly lower. The sensitivity of endothelium-dependent relaxation was significantly impaired in GDM mice (25). Throughout the trimesters, uterine arteries in GDM pregnancies underwent significant changes, and further studies are required to validate these findings. Dysfunction of the uterine artery impairs the utero-placental perfusion and the fetal development. It is still an important question regarding the relationship between uterine artery functions/dysfunction and fetal body weight/growth restriction in GDM.

#### 2.1.4 Carotid artery

In most clinical studies, carotid-femoral pulse wave velocity was increased in women with GDM both during and after pregnancy (26, 27), indicating aggravating arterial stiffness. Women with GDM during pregnancy or a history of GDM had increased carotid intima-media thickness (CIMT), a recognized surrogate marker for future CVD and subclinical atherosclerosis (28, 29). Endothelial function parameters, including the pressure-strain elasticity coefficient, the common carotid stiffness index (β), and the augment index of bilateral common carotid arteries, were significantly elevated in GDM pregnancies, whereas arterial compliance was significantly lower in these patients (27, 30). No significant postpartum differences were observed in the β value and carotid elasticity between the two groups. Overall, the distensibility and elasticity of carotid artery were significantly lower in women with GDM and post-GDM women (31), suggesting an increased risk of subclinical atherosclerosis and stroke (32).

#### 2.1.5 Ocular artery

The vessel density in the central fovea of the superficial and deep retina was remarkably lower in GDM gravidae (33). The central retinal venous diameter was higher, but the artery-to-vein ratio was lower in GDM pregnancies near term (33). The maximum diastolic velocity was significantly higher, while the resistance index was lower in the ophthalmic arteries of women with GDM at GW 28–32 (34). At GW 35–37, women with GDM have been shown to have significantly higher peak systolic velocity ratio in the ophthalmic artery (14). However, some studies reported no significant differences in ophthalmic artery indices in women with GDM, such as the peak systolic velocity ratio delta, pulsatility index, resistance index, peak velocity ratio, peak systolic velocity, and end-diastolic velocity (10, 35). The inconsistent results might be attributed to individual differences (such as GWs and metabolic status) and variations in sample sizes.

#### 2.1.6 Other arteries

The augmentation index, assessed at brachial and radial arteries, has shown inconsistent patterns in women with current or previous GDM. While some studies reported significantly increased augmentation index values, others demonstrated no significant changes compared to healthy controls (21, 36). Pulse wave velocity in the upper limb and aorta was significantly higher in GDM pregnancies at GW 11–13 (21) and in post-GDM women at 5-year follow-up (37). Distensibility of brachial artery was lower in the women with GDM history (31). While some studies reported comparable flow-mediated dilation between GDM and normal pregnancies (38), others have identified lower flow-mediated dilation in post-GDM women compared to the control group (31, 39). Additionally, in GDM mice, maximal endothelium-dependent relaxation was decreased in mesenteric arteries (25). In hypercaloric diet-induced GDM rat, contractile response was impaired, accompanied by altered protein expression of angiotensin type 1 and 2 receptors and cyclooxygenase in the aorta (40). These findings indicate that women with GDM exhibit increased arterial stiffness and impaired vascular function, which may contribute to the increased risk of preeclampsia.

In summary, current evidence strongly supports a significant association between GDM and increased CVD risk in women, including hypertension, coronary artery diseases, atherosclerosis, and subclinical cardiac dysfunction.

### 2.2 Cardiovascular changes in the progeny

The Barker hypothesis, also known as the developmental origins of health and disease theory, proposes that adverse factors *in utero* significantly increase risks of CVDs in the offspring (41). Compared to the control group, GDM fetuses are exposed to higher blood glucose *in utero*, and GDM offspring have been shown an increased risk of congenital heart disease (42) and other CVDs (43). Maternal diabetes during pregnancy increases the rates of early onset of CVDs, particularly hypertension in offspring. GDM exerts long-term effects on offspring blood vessels (cerebral artery, carotid artery, pulmonary artery, aorta, mesenteric artery, and other arteries), both structurally and functionally.

#### 2.2.1 Heart

The majority of studies have demonstrated that fetuses and neonates exposed to GDM had reduced mitral E/A ratio, increased interventricular septal thickness, elevated myocardial performance index, and prolonged isovolumic relaxation time and isovolumic contraction time (44–46). Structural cardiac alterations increased the risk of developing hypertrophic cardiomyopathy and contributed to cardiac systolic and diastolic dysfunction in GDM offspring (47–49). Furthermore, emerging evidence suggests that the right ventricle was more impaired than the left ventricle in GDM offspring (47, 49–51). The right ventricular predominance might be a potential early marker for detecting fetal cardiac dysfunction (48). However, a few studies reported no significant alteration in left ventricular systolic function, myocardial performance index, or E/A ratio in fetuses exposed to GDM (46). The impact of GDM on fetal heart rate remains inconsistent, with reports of both unchanged and increased rates (45, 52). Notably, one study proposed that during the first trimester, fetal heart rate might be highly predictive of GDM (53).

#### 2.2.2 Blood pressure

GDM offspring had higher prevalence of hypertension (43, 54). Children exposed to GDM *in utero* had elevated systolic blood pressure from 3 years of age (43, 54–57), rather than during the first year of life (58). Streptozotocin diabetes in the pregnant animals resulted in hypertension in adult offspring, with elevated blood pressure from 24 weeks of age and persisting elevated throughout 30 weeks (59). The association between GDM and higher blood pressure remained solid only in male offspring (55), and not in female offspring (60, 61). These findings collectively suggest that GDM-related hypertension in offspring is both age-dependent and sex-dependent.

#### 2.2.3 Cerebral artery

Fetus exposed to GDM demonstrates the hemodynamic alterations, with studies reporting decreased peak systolic velocity in middle cerebral arteries (24, 62, 63) and no significant changes (64). The complicated results were observed in other hemodynamic indices, such as systolic/diastolic ratio, resistance index, and pulsation index (24, 64, 65). Comparative studies reveal that children aged 9–11 years with GDM exposure had increased hypothalamic blood flow (66). Maternal high-sucrose diets consumption during pregnancy induced alterations in cerebral artery function in offspring (67).

#### 2.2.4 Carotid artery

As a primary conduit for cerebral blood supply, the carotid artery exhibits significantly structural and functional changes in GDM progeny. Following intrauterine exposure to GDM, CIMT in neonates was increased or unchanged (68–70). Higher levels of fasting plasma glucose at 26 weeks of gestation were strongly related to increased CIMT in their offspring at the age of 6 years (71).

#### 2.2.5 Pulmonary artery

Maternal hyperglycemia inhibited pulmonary vasculogenesis during fetal development (72). During pregnancy, there was no significant difference in acceleration time and acceleration time/ejection time (73). At 1 year of age, the acceleration time of the pulmonary artery in children born to GDM mothers was significantly lower (74).

#### 2.2.6 Aorta

Previous studies found that in human fetuses exposed to GDM, the propagation velocities of the aortic arch were reduced at GW 34–40 (75). In 3- to 5-day-old human infants born to mothers with diabetes, the intima media thickness of the abdominal aorta was increased, while in 1-year-old offspring of women with GDM, it was not (58, 76). There was an increased aortic augmentation index in the GDM offspring (71). When compared with the aorta of the control offspring, KCl-, endothelin-1-, and noradrenaline-mediated vasoconstriction was potentiated and acetylcholine-mediated vasodilation was reduced in streptozotocin-induced female offspring but not in the male offspring, indicating that GDM programs gender-specific vascular dysfunction in the aorta (77).

#### 2.2.7 Mesenteric artery

The mesenteric vasculature is closely associated with blood pressure, as it constitutes a component of systemic resistance arteries. Offspring of maternal diabetes during pregnancy showed an impaired endothelium-dependent relaxation in mesenteric arteries (25, 59), whereas relaxation to sodium nitroprusside remained unchanged (78). Adult offspring exposed to maternal diabetes during pregnancy had enhanced sensitivity to noradrenaline (78). Maternal high-sucrose diets accelerated vascular stiffness in the aged offspring, characterized by weakened myogenic responses and reduced phenylephrine-stimulated contraction (79).

#### 2.2.8 Renal arteries

Doppler ultrasound analysis revealed that the systolic/diastolic ratio, resistance index, and pulsatility index were increased in the renal artery of GDM fetuses (65, 80). Neonates of mothers who maintained strict normoglycemia control during pregnancy and met the other criteria of the GDM management program exhibited no changes in renal volumes, urinary biomarkers of renal functions, or markers of tubular impairment compared to the control group. Conversely, neonates of mothers who did not maintain glycemic control and were non-compliant with the management program exhibited significantly lower renal volumes and higher activities of N-acetyl-β-D-glucosaminidase and cathepsin B (81).

#### 2.2.9 Others

High glucose exposure during pregnancy inhibited the development of the blood vessel plexus and resulted in narrower blood vessel diameter in chick embryo (82).

In conclusion, GDM has been shown to exert both short-term and long-term effects on offspring circulation, which may be age-dependent and gender-specific. The development of CVDs in GDM offspring may be attributed to maternal hyperglycemia. Thus, glycemic control during pregnancy is vital for the cardiovascular health of GDM offspring.

### 2.3 Umbilical–placental circulation

Umbilical–placental circulation is essential for material exchange between the mother and fetus, typically comprising one umbilical vein, two umbilical arteries, stem placental villi, intermediate villi, and terminal villi. In normal pregnancies, the structure of chorionic villi ensures proper nutrient delivery to the fetus.

GDM is a pathology associated with vascular dysfunction in umbilical–placental circulation. In GDM, placental villi exhibit hypoplasia, with immature villi, abnormal villi branching, and excessive neovascularization (83). It is characterized by an increased distance between the intervillous space and fetal capillaries in the GDM placenta (84). The microvilli of the GDM placenta were disorganized and locally hyperplastic, with some areas showing sparse or even absent microvilli (85). The endoplasmic reticulum and mitochondria of trophoblast cells were significantly swollen, the basement membrane was thickened, and there were varying degrees of hyperplasia in the small placental arteries (83). The barrier integrity of placental vessels was compromised in GDM (86). The structural alterations in placental blood vessels seriously impair blood and oxygen supply between the placenta and fetus, and may be one of the key factors contributing to adverse pregnancy outcomes in GDM (87).

Bahiru et al. reported histopathologic changes in GDM, including umbilical cord crack, disintegration of the endothelium, and crack of umbilical vessels. Endothelial cells in GDM umbilical cords were discontinuous with focal erosions (88). Smooth muscles of GDM umbilical blood vessels appeared disturbed and showed degeneration of their strands (89). The media of GDM umbilical artery showed smooth muscle cells widely separated by connective tissue containing little collagen and few elastic fibers, along with mononuclear cell infiltration. The GDM umbilical vein has a thinner wall and a wider lumen (90).

Alterations in umbilical–placental vessel structures are closely associated with blood flow and vascular tone. Pregnancies complicated by GDM exhibited significantly lower placental volume, vascularization index, and vascularization flow index in the placenta compared to the control group during the first and second trimesters (23, 91). Most studies found that hemodynamic indices of the GDM umbilical artery, such as peak systolic velocity/end-systolic blood flow velocity, resistance index, and pulsation index, were reduced in the third trimester (65, 92). However, Cui et al. reported higher peak systolic velocity/end-systolic blood flow velocity, resistance index, and pulsation index values in the GDM umbilical artery during the third trimester, and also significantly lower peak systolic and minimum diastolic velocities (24). GDM was also reported to have no association with abnormal Doppler indices of placenta circulation (93). One possible explanation for that discrepancy is the existence of individual differences and the varying levels of maternal hyperglycemia.

Miroslav et al. found that in the GDM umbilical artery, cumulative concentrations of 5-HT-mediated vasoconstrictions were significantly attenuated (94), and the concentration–response curve for bradykinin was shifted to the left after endothelial denudation (95). Omar et al. noted that placental vasodilation caused by progesterone via cyclic adenosine monophosphate was significantly reduced (96). Abnormal vessel tone of the umbilical–placental circulation might decrease placental perfusion and the blood flow to the fetus.

Abnormal umbilical–placental circulation in GDM might be one of the most important reasons for cardiovascular changes in progeny and in women.

## 3 Mechanisms in GDM-related cardiovascular changes

Cardiovascular changes in women with GDM and their offspring were correlated with endothelial dysfunction, insulin resistance, oxidative stress, ion channels, inflammation, angiogenesis, and epigenetic inheritance. These mechanisms could be crucial for the better management of cardiovascular changes in GDM.

### 3.1 Endothelial dysfunction

Endothelial dysfunction is considered to be a hallmark of vascular disorders. Endothelial dysfunction is widely observed in GDM pregnancies, post-GDM women, the umbilical–placental circulation, and their offspring. Thus, it could be one of the mechanisms underlying GDM-induced CVDs.

GDM pregnancy exhibited impaired endothelium-dependent relaxation to methacholine in mesenteric arteries (25), along with decreased circulating endothelial progenitor cell counts (97), and modified endothelial function markers, such as nitric oxide (NO) and endothelial nitric oxide synthase (eNOS). Reduced bioavailability of NO is a consensus among researchers studying GDM (98). Women with previous GDM displayed lower flow-mediated dilation (39), higher values of markers of endothelial dysfunction, such as E-selectin and intercellular adhesion molecule-1 (ICAM-1) (99), and decreased levels of L-arginine (a critical substrate for NO synthesis) (100). These findings indicated that GDM-related endothelial dysfunction could persist into postpartum.

The umbilical–placental circulation is considered to be part of fetal circulation. Endothelial rupture and erosion were observed in umbilical vessels from GDM pregnancies (88). Fetal endothelial progenitor cells exposed to hyperglycemia *in vivo* or *in vitro* formed fewer colonies in culture, and displayed reduced proliferation, migration, and tubule formation (101). Endothelium-dependent relaxation to calcitonin was weaker in GDM umbilical veins than that in the control group (102). When compared to the control group, NO synthase activities were decreased in GDM stem villous vessels (103). The offspring from mothers with diabetes exhibited impaired endothelium-dependent relaxation in mesenteric arteries (59), decreased NO production and lowered eNOS phosphorylation in blood vessels (104), and reduced eNOS functions in regulating vessel tone (105). Thus, it is suggested that GDM-related endothelial dysfunction in the progeny may originate from the prenatal period.

However, there were significantly higher circulating endothelial functional and dysfunctional markers, including von Willebrand factor and eNOS, in GDM umbilical plasma (75, 97). In primary feto-placental endothelial cells from GDM pregnancies, there was a decrease in ICAM-1, a marker of endothelial dysfunction (106). Human umbilical vein endothelial cells (HUVECs) from GDM pregnancy or HUVECs exposed to hyperglycemia showed significantly increased L-arginine transport, enhanced human cationic amino acid transporter-1, and eNOS expression and activities (98, 107, 108). The inconsistent findings may be attributed to different tissues studied and different levels of glycemic control.

The increased circulating endothelial functional markers could originate from the umbilical–placental endothelium. Exosomes isolated from HUVECs of normal pregnancies could inhibit the changes in HUVECs from GDM pregnancies mentioned above. Conversely, exosomes from GDM HUVECs reduced eNOS phosphorylation and increased reactive oxygen species (ROS) generation in cells from normal pregnancy (108). Insulin could reverse GDM-related endothelium abnormalities (109) via activation of insulin receptors (110), A1 adenosine receptors (109), and nicotinamide adenine dinucleotide phosphate (NADPH) oxidase (111). The inhibition of endoplasmic reticulum stress and reduction of ROS levels could increase NO production and restore endothelium-dependent vasodilation in offspring of mothers with diabetes (59, 104).

### 3.2 Insulin resistance

**Figure 1:**
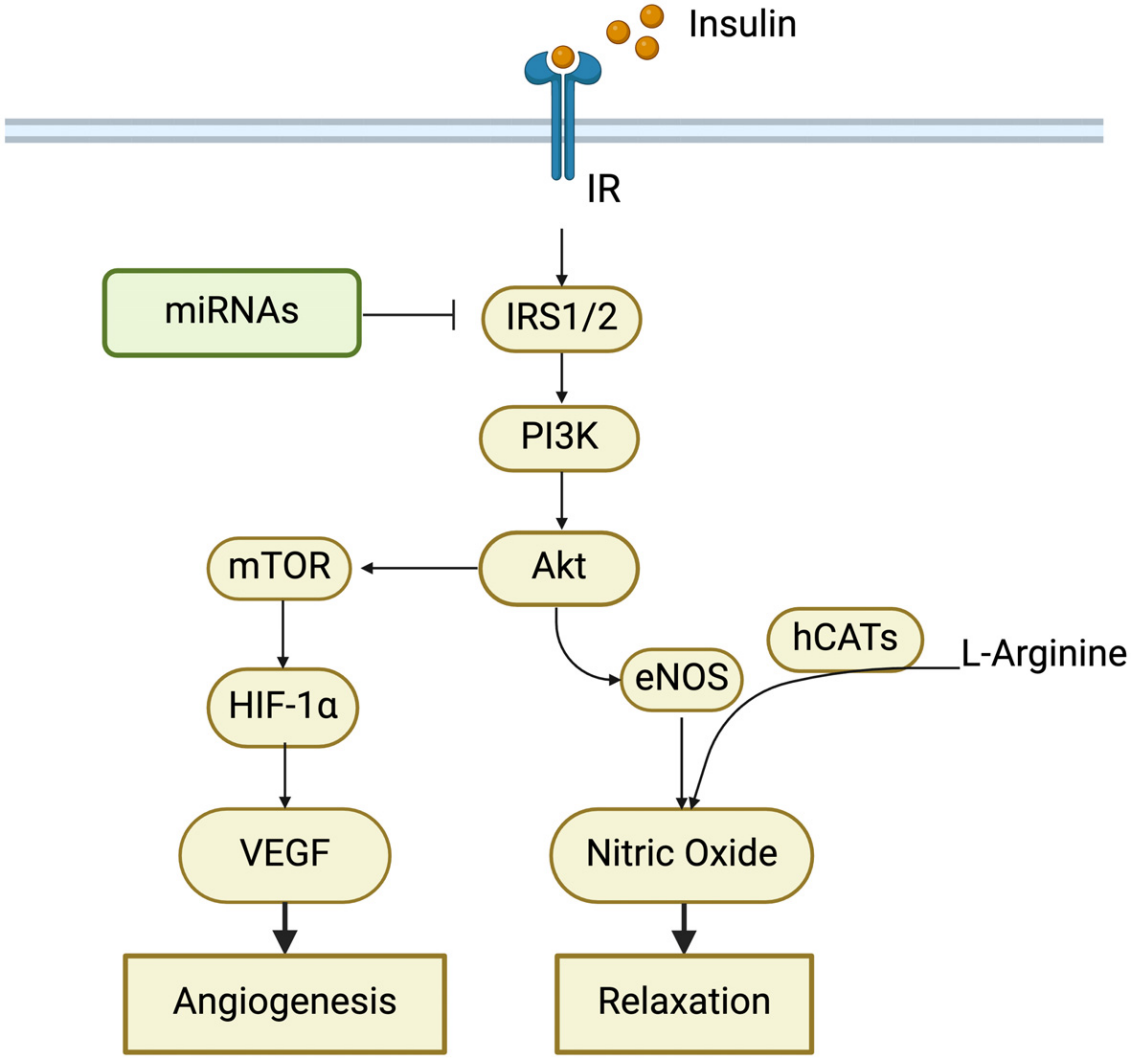
Role of insulin signaling pathway in cardiovascular changes in GDM. Alterations in insulin receptors (IR), insulin receptor substrate proteins 1/2 (IRS1/2), and PI3K-Akt-mTOR pathway contribute to insulin resistance, which has been observed in women with GDM and their offspring. MicroRNAs (miRNAs) can regulate insulin signaling by targeting IRS1/2. Insulin resistance impairs angiogenesis and endothelial dilation, thereby increasing the risk of developing cardiovascular diseases in GDM.

Insulin resistance is a pathophysiological condition in which organs do not respond appropriately to insulin, observed in GDM pregnancies, post-GDM women, and GDM offspring, and the GDM umbilical–placental circulation (77, 112–116). Changes in insulin signaling pathways (**Figure 1**), such as insulin receptors and substrates, MAPK, JNK, PI3K, AKT, and mTOR, contribute to insulin resistance in GDM (117). In the plasma of women with GDM pregnancy and their offspring, there were alterations in insulin resistance-related factors, such as elevated leptin (118), tumor necrosis factor-α (TNF-α), asprosin (116), and resistin (119). The GDM placenta had increased levels of glucose transporter-4 and glucose transporter-8, and decreased levels of glucose transporters-3, which were one of the mechanisms of insulin resistance (120).

Flow-mediated dilation in brachial arteries in women with previous GDM was correlated inversely with serum markers of insulin resistance (39). Insulin resistance was found to be associated with vascular dysfunction (especially endothelium dysfunction) and arterial stiffness (121–124), thereby increasing the risks of developing CVDs. Increased maternal insulin resistance had a negative impact on placental efficiency in GDM cases (125), which may be due to the expansion of immature villi (126). Astaxanthin and naringenin have the potential to attenuate GDM symptoms by improving insulin sensitivity during pregnancy through adenosine 5′-monophosphate-activated protein kinase (127, 128).

### 3.3 Oxidative stress

Oxidative stress increases during gestation, and the placenta is considered to be the primary source of ROS generation (129). In the offspring and maternal tissues of GDM pregnancies, there were increased markers of oxidative stress, such as higher levels of circulating free radical production in the mothers and offspring, and reduced catalase activity in the placenta and fetus (59, 130–132).

Maternal hyperglycemia is regarded as an important cause of oxidative stress in GDM. Hyperglycemia contributes to increased ROS synthesis in endothelial cells. HUVECs from GDM showed an increased ROS synthesis, and HUVECs from normal pregnancies exposed to a high extracellular concentration of D-glucose increased NOX-dependent ROS generation (133, 134). Hyperglycemia stimulated ROS production through glucose autoxidation, mitochondrial superoxide production, eNOS uncoupling, and late glycosylation end product-dependent NADPH oxidase activation (135).

**Figure 2:**
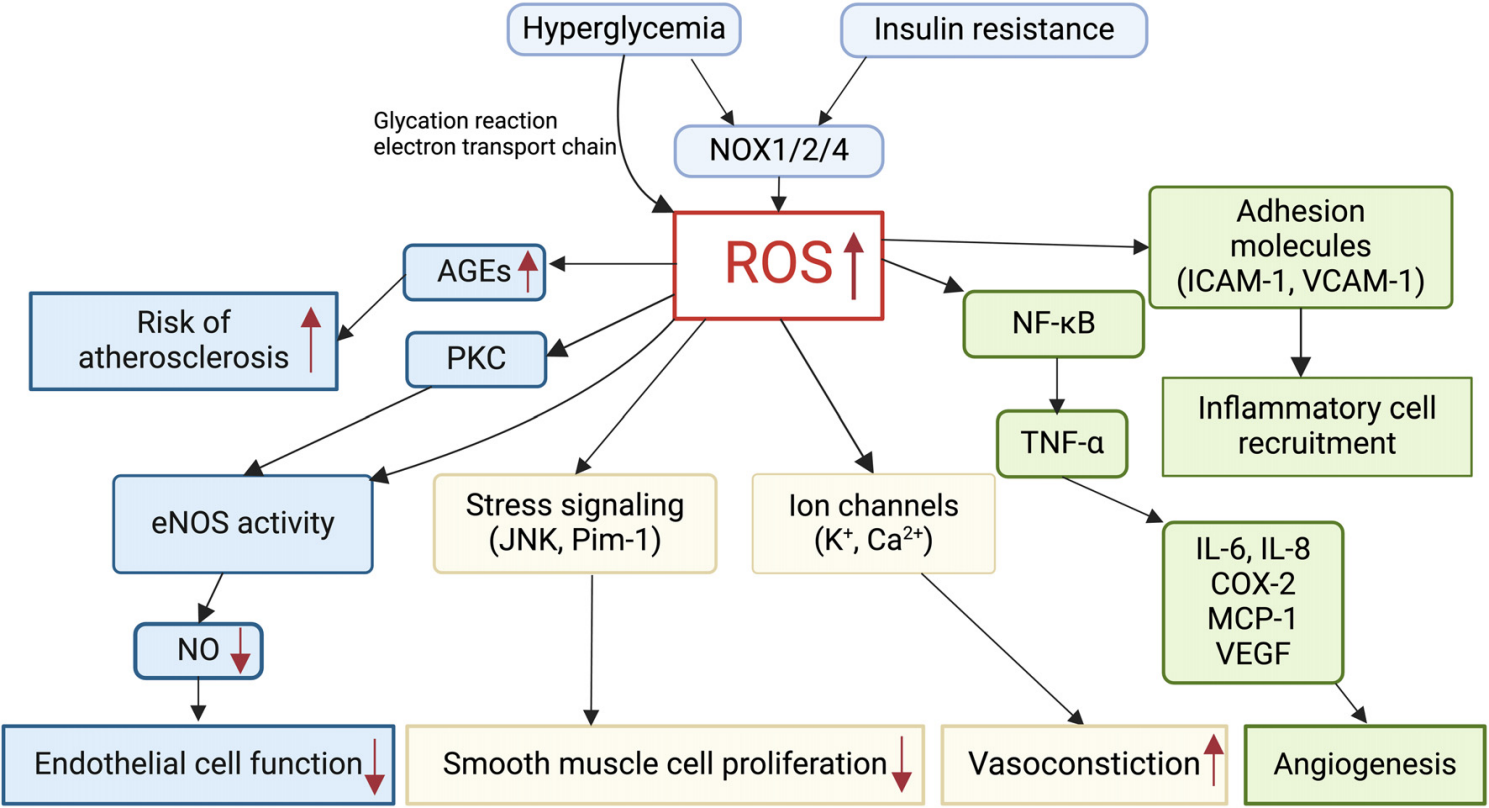
Role of reactive oxygen species (ROS) in cardiovascular alterations in GDM. Hyperglycemia and insulin resistance induce excessive ROS production in GDM via NADPH oxidase (NOX). Elevated ROS levels in GDM impair endothelial cell function, smooth muscle cell proliferation, vasoconstriction, and angiogenesis. ROS mediates the production of advanced glycation end products (AGEs), thereby increasing the risk of atherosclerosis. Furthermore, ROS promotes inflammatory cell recruitment and inflammation in GDM.

Oxidative stress in GDM pregnancy could increase cardiovascular risks in the mother and fetus, via endothelial dysfunction, decreased NO bioavailability and inflammation, and altered ion channel activities (**Figure 2**). ROS increased 4-hydroxynonenal production and damaged the development of coronary artery in pre-gestational diabetes fetus (136). Increased ROS and NADPH activities might cause endothelial dysfunction via the protein kinase C pathway in GDM mothers and their fetuses (137). ROS was found to induce an increase in inflammatory factors, such as interleukin-6 (IL-6) and TNF-α, which were implicated in GDM placental vascular endothelial dysfunction (138, 139). In the offspring exposed to maternal hyperglycemia, NOX4-derived superoxide inhibited large-conductance Ca^2+^-activated potassium channel (BK_Ca_) activities via the AKT pathway (140). ROS affected transient receptor potential (TRP)-type Ca^2+^-permeable non-selective cation channels by targeting both membrane lipids and channel proteins in the term syncytiotrophoblast (141). ROS increased the expressions of multiple growth factors and activated multiple stress signals such as JNK and Pim-1, leading to smooth muscle cell proliferation, and regulated angiogenesis through NF-κB/TNF-α signaling pathway and related factors such as IL-6, ICAM-1, and vascular endothelial growth factor (VEGF) (142).

### 3.4 Ion channels

Cation channels, such as K^+^ and Ca^2+^ channels, play an important role in the regulation of vessel tone. Li et al. demonstrated that ATP-sensitive potassium channel (K_ATP_) currents and K_ATP_ channel-mediated relaxation were impaired in GDM umbilical arteries (143). Djokic et al. found that a K^+^ channel opener, pinacidil, reduced relaxation in endothelium-denuded HUV compared to those from normal pregnancy, while the expression of K_ATP_ channels was decreased in GDM umbilical veins (144). BK_Ca_ current density in human umbilical artery smooth muscle cells was significantly reduced, and vasodilation mediated by BK_Ca_ agonist NS-1619 was significantly impaired in GDM (145). Changes in inwardly rectifying potassium channel and small-conductance calcium-dependent potassium channel were associated with attenuated bradykinin-mediated contraction in GDM umbilical arteries (95). Some studies reported that polymorphisms of KCNQ1 (rs2237892, rs2237895, and rs2074196) and KCNJ11 (E23K) were associated with GDM (146–149), but others found that gene polymorphisms of KCNJ11 (rs5219) and KCNQ1 (rs2237892, rs151290, rs231841, and rs7929804) were not significant risk factors for the development of GDM (150–152).

Heather found that the reduced rate of Ca^2+^ bursting in GDM umbilical vein endothelial cells inhibited the functions of NO, thereby leading to vascular dysfunction (153). Moreover, Miroslav et al. found that serotonin-mediated vasoconstriction was significantly attenuated in GDM umbilical arteries, which was associated with the impairment of voltage-gated Ca^2+^ channels and Na^+^/K^+^-ATPase (94). Furthermore, in GDM placenta, mRNA expressions of calcium transporters were downregulated, including TRPV5 and TRPV6, calcium-binding/chaperone proteins, plasma membrane calcium ATPase, inositol triphosphate receptors, and ryanodine receptors (154).

Maternal high-glucose diets during pregnancy altered the frequency and amplitude of BK_Ca_ channels, as well as L-type voltage-dependent Ca^2+^ channel currents in the offspring vasculature (155). Hyperglycemia affected the activities of ion channels in vascular smooth muscle (156). Altered functions, expressions, and polymorphisms of ion channels could contribute to the increased risks of developing CVDs in GDM. The role of ion channels in GDM has primarily been studied in human umbilical and placental vasculature, which is functionally analogous to fetal vasculature and provides insights into offspring cardiovascular programming.

### 3.5 Inflammation

Inflammatory factors play a key role in the process of GDM and GDM-mediated vascular changes. Many inflammatory factors are closely linked to GDM-mediated vascular injury, including C-reactive protein (CRP), ICAM-1, vascular cell adhesion molecule-1 (VCAM-1), and IL-6, among others (157).

Many studies demonstrated that CRP was increased in maternal serum, cord serum, and the placenta of women with GDM (158–161). Higher circulating CRP may predict the risk of GDM development. CRP was one of the significant independent predictors of developing preeclampsia in women with GDM (162). Insulin administration significantly reduced CRP concentration and ameliorated aortic injury in streptozotocin-mediated diabetic rats (163).

ICAM-1 was viewed as a symbol of endothelial dysfunction leading to vascular disorders, and its level was increased in GDM maternal serum and the umbilical–placental circulation (98, 164). Exposure to high glucose could enhance ICAM-1 expression in HUVECs by increasing the release of exosomes (165). Increased ICAM-1 significantly promoted monocyte adhesion to decidual endothelial cells in diabetic pregnancies, which could be inhibited via ICAM-1 silencing (166). In GDM umbilical cords and placental vessels, the immunostaining intensity of ICAM-1 was decreased compared to the control group (164). ICAM-1 protein was lower in primary feto-placental endothelial cells from GDM pregnancy when compared with the control group (106). Decreased ICAM-1 caused by elevated miR-130b-3p from GDM-placenta mesenchymal stem cell-derived exosomes participated in the inhibition of HUVEC proliferation, migration, and angiogenesis (167). Altered ICAM-1 plays an important role in GDM vascular pathology.

VCAM-1 was increased in the maternal serum, umbilical cord, and placenta of patients with GDM (98, 168, 169). After delivery, circulating VCAM-1 remained increased in women with GDM (170). VCAM-1 mRNA and protein levels were unchanged in primary feto-placental endothelial cells from GDM pregnancy when compared with the normal group (106), although other reports showed that VCAM-1 was increased in GDM placenta (171). High glucose stimulated the expression of VCAM-1 in HUVECs (168). Previous studies demonstrated that increased ICAM-1 and VCAM-1 were the first critical step for lymphocyte and endothelial cell interactions (172). Increased VCAM-1 primed diabetic vasculature to have enhanced interaction with circulating monocytes in human endothelial cells cultured with advanced glycation end products (173).

In GDM maternal blood and umbilical cord blood, IL-1β and IL-6 were increased or unchanged (174–178). Moreover, on the third day postpartum, women with GDM were found to have higher circulating IL-1β levels (176). Additionally, GDM placenta showed increased IL-1β and IL-6 expression (159, 179). Increased IL-1β and IL-6 were associated with vascular dysfunction in retinal arteries (180) and in forearm skin vessels (181) from GDM pregnancies. The interaction of IL-6 and TNF-α contributed to endothelial dysfunction in diabetic mice via oxidative stress and reduced eNOS phosphorylation (182). There were decreased IL-37 in the GDM umbilical–placental system (183). IL-37 inhibited the progression of vascular calcification and atherosclerosis in diabetes (184). There were also many changes in inflammatory factors in GDM, such as TNF-α, IL-10, IL-8, and IL-38. Inflammatory factors could affect endothelial functions and vascular calcification, which might finally lead to vascular disease in GDM. More studies are needed to clarify the role of inflammatory factors in GDM vascular dysfunction.

### 3.6 Angiogenesis

Angiogenesis is a coordinated process of proangiogenic and inhibitory factors. Histopathological analysis indicates excessive angiogenesis in GDM placenta, including increased villous vascularity and elevated number of syncytial knots (185). There were commonly increased proangiogenic factors, including the VEGF-signaling pathway (186, 187), total and active membrane-type matrix metalloproteinase 1 (188), and cognate succinate receptors (189) in GDM placenta, but there was a reduction of anti-angiogenic receptor UNC5b in GDM HUVECs (190). GDM-derived trophoblast showed altered expressions of proangiogenic factors and anti-angiogenic factors (138). Hyperglycemia-induced angiogenesis changes were associated with molecules in trophoblast (191). Exposure to GDM-like conditions enhanced the proangiogenic abilities of human amniotic membrane stem cells (192).

However, some studies reported that when compared with the control group, there was a decrease in angiogenic factors and angiogenesis modulators, such as SIRT1 (193), VEGFA, and VEGFR2 in GDM placenta (194). Maternal hyperglycemia inhibited angiogenesis in fetal pulmonary arteries (72). The HUVECs from GDM pregnancies presented increased apoptosis and decreased proliferation and angiogenesis compared with those from healthy pregnancies (195). Both the GDM conditions and hyperglycemia inhibited HUVEC proliferation, migration, and tube formation via reduced FGF2-induced activation of ERK1/2, and caused apoptosis via increased calcium entry (196, 197).

Alterations in angiogenesis in GDM were closely associated with maternal hyperglycemia, which might lead to abnormal development of both the placenta and the fetus. Abnormal umbilical coiling in GDM was related to the downregulation of the angiogenic factor VEGFA (198). The inconsistent findings may be attributed to variations in tissue types and differences in GDM-like conditions. Therefore, further studies are needed to clarify the mechanisms.

### 3.7 Epigenetic modification

Epigenetic mechanisms, including DNA methylation, microRNAs (miRNAs), long noncoding RNAs (lncRNAs), and histone modifications, can produce heritable phenotypic changes without altering the DNA sequence.

DNA methylation is widely observed in GDM (199, 200). Exposure to GDM has been shown to alter DNA methylation patterns in human feto-placental arterial and venous endothelial cells, leading to aberrant cellular morphology and impaired barrier function in endothelial cells (201, 202). Notably, the promoter region of estrogen receptor 1 was found to be methylated in decidual vessels of healthy individuals, but not in GDM (203). DNA hypermethylation of HDAC2 was significantly more pronounced in GDM-HUVECs compared to control-HUVECs (204). Sun et al. further demonstrated that the abundance of 5-hydroxymethylcytosine (5hmC) in the umbilical vein of women with GDM was altered, a change linked to DNA methylation-related plasticity through oxidation mediated by ten-eleven translocation enzymes (205). These alterations in DNA methylation and 5hmC levels in GDM reflected the molecular characteristics of “type II diabetes” and “insulin resistance,” contributing to abnormal cardiovascular development and an increased risk of cardio-metabolic diseases later in life.

Multiple miRNAs are reported to play roles in cardiovascular changes associated with GDM. In a GDM rat model, inhibition of miR-873, which targeted IGFBP2, was shown to regulate insulin resistance and alleviate myocardial injury by activating the PI3K/AKT/mTOR signaling pathway, thereby mitigating the progression of GDM (206). Additionally, decreased levels of placenta-derived exosome miR-140-3p and miR-574-3p in GDM were found to inhibit the proliferation, migration, and tube formation capacity of umbilical vein endothelial cells by targeting VEGFs (207). However, miR-130b-3p exhibited an opposite effect on HUVECs compared to miR-140-3p and miR-574-3p, as its upregulation inhibited HUVEC proliferation and angiogenesis (167). Alterations in cerebrovascular functions in GDM offspring may be attributed to changes in miR-29a-3p and miR-92a-3p levels (208). Although a large number of differentially expressed miRNAs have been identified, further research is needed to elucidate the relationship between miRNAs and cardiovascular changes in GDM (209).

In GDM pregnancies, changes in circulating lncRNAs have been observed, including decreased lncRNA SNHG17 and increased lncRNA SOX2OT, which were strongly associated with adverse outcomes such as intrauterine distress and hypertension (210). Elevated levels of circVEGFC in maternal serum from GDM pregnancies might be linked to hypertension (211). Furthermore, high sucrose intake upregulated angiotensin 1 receptor expression through histone modifications, such as increased H3Ac, H3K4me3, and H3S10ph, as well as decreased H3K9me3, ultimately contributing to hypertension in aged offspring (156).

Epigenetic mechanisms may serve as mediators of persistent metabolic memory in endothelial cells exposed to hyperglycemia (204). These epigenetic modifications affect insulin resistance, angiogenesis, and vascular functions, finally leading to cardiovascular changes in GDM.

## 4 Prevention and treatment of GDM-related cardiovascular diseases

GDM significantly increases cardiovascular risks in both mothers and offspring. Insulin and metformin are commonly used to treat GDM, improving immediate pregnancy outcomes, and reducing the incidence of pregnancy-related hypertension (212). Metformin treatment in GDM pregnancy is associated with a reduced risk of preeclampsia (213). Additionally, treatment with metformin alone or in combination with insulin has been shown to ameliorate the increased augmentation index in the brachial arteries and aorta during GW 28–36 in GDM pregnancies (214). Insulin-treated GDM pregnancies exhibited a resistance index of umbilical arteries similar to that of the control group (92). This review summarizes the effects of exercise, dietary modification, and probiotics on cardiovascular changes associated with GDM.

### 4.1 Exercise

Exercise is effective for controlling blood glucose and insulin levels in GDM pregnancies (215, 216). It modestly improved cardiorespiratory fitness in both GDM pregnancies and their fetuses (217), as evidenced by elevated heart rates (218). Moderate-intensity resistance exercise has been found to be beneficial for improving blood pressure in patients with GDM (219). Exercise reduced uterine artery pulsatility indexes in GDM pregnancies (220). During exercise, women with GDM exhibited blunted cerebral oxygenation, which was correlated with macrovascular functions (221). Moderate-intensity exercise improved oxidation capacity in GDM pregnancies (220). Exercise is highly recommended for the management of GDM and has been shown to be beneficial in preventing cardiovascular damage in both mothers and their offspring.

### 4.2 Dietary modification

Modified dietary interventions favorably influenced maternal glycemia, insulin levels, and fetal birth weight in GDM (222, 223). Compared with the control group, there was an increased augmentation index in the brachial arteries and aorta from GDM pregnancies with diet management, which could be attenuated by treatment with metformin alone or in combination with insulin (214). The resistance index in umbilical arteries was lower in GDM pregnancies managed with diet interventions compared to the control group, whereas no significant difference was observed between the insulin-treated GDM group and the control group (92). In diet alone-controlled GDM placentas, occludin expression was lower than that in placentas from normal pregnancies and metformin-controlled GDM pregnancies (86). These findings indicated that dietary modification alone during pregnancy may not be sufficient to reverse impaired vascular functions and placental barrier integrity. However, a diet rich in monounsaturated fatty acids demonstrated favorable effects on diastolic blood pressure in women with GDM compared to a high-carbohydrate diet (224).

### 4.3 Probiotics

The consumption of *Lactobacillus* and *Bifidobacterium* probiotics decreased fasting plasma glucose, serum insulin levels, insulin resistance, inflammatory factors (such as CRP and IL-6), and oxidative stress markers, while probiotics significantly increased insulin sensitivity, plasma NO levels, and total antioxidant capacity in GDM pregnancies (225–227). However, some studies have reported that probiotic supplementation was not associated with a reduced risk of hypertensive disorders in GDM pregnancies (228, 229). In contrast, excessive probiotic supplementation might increase the risk of preeclampsia in women with GDM (230). The inconsistent findings may be attributed to variations in probiotic strains, dosages, timing of intervention, and individual differences. Probiotics have demonstrated a positive impact on glycemic control, and further research is needed to clarify their role in preventing CVDs in women with GDM and their offspring.

## 5 Conclusions

GDM exerts both short- and long-term effects on cardiovascular changes in mothers and their offspring. The influence of GDM on offspring may stem from alterations in umbilical–placental circulation and the direct consequences of maternal hyperglycemia. Endothelial dysfunction, insulin resistance, oxidative stress, ion channel abnormalities, inflammation, impaired angiogenesis, and epigenetic modifications collectively contribute to the structural and functional abnormalities of the cardiovascular system in GDM. Early diagnosis and intervention, along with strategies such as exercise, dietary modifications, and probiotics supplementation, may have beneficial effects on GDM-related cardiovascular changes.
